# Testicular Cancer mortality in Brazil: trends and predictions until 2030

**DOI:** 10.1186/s12894-019-0487-z

**Published:** 2019-07-05

**Authors:** Samara Carollyne Mafra Soares, Katarina Márcia Rodrigues dos Santos, Fabia Cheyenne Gomes de Morais Fernandes, Isabelle Ribeiro Barbosa, Dyego Leandro Bezerra de Souza

**Affiliations:** 1 Student in the Graduate Program in Collective Health, Health Science Faculty of Trairi, Federal University of Rio Grande do Norte (UFRN), Natal, Brazil; 2 Undergraduate student in Nursing, Health Science Faculty of Trairi, Federal University of Rio Grande do Norte (UFRN), Natal, Brazil; 3 Graduate Program in Collective Health, Universidade Federal do Rio Grande do Norte/Federal University of Rio Grande do Norte. Programa de Pós-Graduação em Saúde Coletiva, Avenida Senador Salgado Filho 1787, CEP: 59010-000 Lagoa Nova, Natal, RN Brazil

**Keywords:** Mortality, Predictions, Public health, Testicular neoplasm, Time series studies

## Abstract

**Background:**

Despite the fact that testicular cancer presents good prognosis, wide variations in mortality rates have been reported internationally. In Brazil, mortality trends and estimates have not been fully assessed. The objective of the study presented herein is to analyze the mortality trends for testicular cancer in Brazil in the period 2001–2015 and calculate mortality predictions for the period 2016–2030.

**Methods:**

This is a population-based ecological study that utilized information of the Mortality Information System, on testicular cancer-related deaths in Brazil. Mortality trends were analyzed by Joinpoint regression, and Nordpred was utilized for the calculation of predictions.

**Results:**

The mortality rate for men, standardized to the world population, varied between 0.36/100,000 for the year 2001, to 0.41/100,000 for the year 2015. There was an increasing trend for Brazil (APC = 1.3% CI95% 0.6; 2.0) and the Southeast region (APC = 1.5% CI95%0.2; 2.7). When analyzing Brazilian data for the period 2016–2030, predictions indicate 2888 deaths due to testicular cancer, which corresponds to a 26.6% change when compared to the 2011–2015 period. This change is mostly explained by an increase in the risk of death (14.2%) when compared with modifications in the demographic structure (12.4%).

**Conclusions:**

Testicular cancer mortality in Brazil presents increasing trends, and until 2030 these rates continue to increase.

## Background

In recent years, testicular cancer incidence has increased, especially in developed countries, where Europe and North America correspond to approximately 50% of the new cases registered in the world, in 2018 (6.2/100,000 men and 5.1/100,000 men, respectively). The age standardized world incidence rate for testicular cancer by world standard population was 1.7/100,000 men, in 2018 [1].

Testicular cancer is considered rare, and usually affects young men (aged between 15 and 39 years) [1]^,^ representing approximately 0.7% of all cancers [2]. Although this type of tumor affects a relatively small share of the population, it affects a productive and sexually active age group, with negative impacts on social and economic aspects. It must be highlighted that this type of cancer is usually aggressive, but presents high chances of cure when detected early [3], justifying the importance of its study to public health.

The age standardized world mortality rate for testicular cancer by world standard population was 0.23/100,000 men. In 2018, world estimates reported the incidence of 71,105 new cases and approximately 9507 deaths, of which only 2131 occurred in the most developed regions of the world [1]. Although the highest incidence rates occurred in more developed regions, approximately 80% of the deaths attributed to testicular cancer occurred in developing countries, with Latin America and the Caribbean presenting very high rates (0.55 deaths/100,000 men) [4].

Testicular cancer presents a strong genetic component in its development, and is 4 to 5 times more prevalent in Causasians than in Afrodescendant populations. It usually affects young men, with the following risk factors: cryptorchidism, infertility, family history (heritability varies between 37 and 49%) and also associated with the Klinefelter syndrome [2].

Testicular tumors are histologically classified in seminoma and non-seminoma. Approximately 70% of diagnosis are made in initial stages, with excellent cure rates due to its high sensitivity to chemotherapy and radiotherapy, especially if detected early [5]. Five-year survival rates also vary by histological type, from 48 to 92%. [6].

Because testicular cancer presents significant mortality in developing countries, comprehension of its geographic distribution and behavior of rates throughout time is very relevant. Analysis of the epidemiological situation is necessary as an instrument to support the planning of public health policies for the most vulnerable groups. There is a lack in the scientific literature on the epidemiology of testicular cancer in South America, especially in Brazil.

The objective of this study was to analyze the temporal trends of testicular cancer mortality in Brazil and its geographic regions between 2001 and 2015 and estimate mortality predictions for the period 2016–2030.

## Methods

An ecological study of temporal series is presented herein, based on secondary data collected prior to this research and available online, from the Mortality Information System (SIM) of the Informatics Department of the Unified Health System (Brazil’s publicly funded health system). Deaths due to malignant testicular neoplasm (C62) were analyzed, categorized from the International Classification of Diseases, 10th Revision (ICD-10) and performed equivalence for the ICD- 9, occurred in Brazil in the period 2001–2015 and analyzed according to age groups and geographic regions (five geographic regions).

According to SIM [7], secondary data at regional level for “Ill-defined causes in cause-of-death registration (%)” and “Civil registration coverage of cause-of-death” are, respectively: North (12.56%/81.21%), Northeast (10.96%/84.1%), Southeast (11.76%/88.05%), South (9.73%/ 91.86%), Midwest (8.43%/ 90.33%). The mortality information system of Brazil and its geographical regions can be classified as presenting intermediate quality [8]. In recent years, although the quality of registries within SIM has improved considerably, the utilization of secondary data on mortality is still subject to under-registry. Information was utilized from the redistribution of deaths by chapter, corrected by active search [9], to account for the under-registry of deaths due to malignant testicular neoplasms. This is an initiative of the Brazilian Ministry of Health, with data made available at the website of the Informatics Department of the Unified Health System (http://datasus.saude.gov.br/informacoes-de-saude/tabnet) [9].

The percentage difference between the amount of notified deaths to SIM and the amount of redistributed deaths, based on chapter II (neoplasms) of ICD-10, was obtained from a correction factor calculated for each age group, time period, and geographic region [10].This difference was expressed in decimal values, with 1 corresponding to a 100% change, for example. Higher values are possible, as the redistributed amount of deaths could present higher values than what was reported to SIM. When the redistributed value was lower than what was reported to SIM, a negative difference was obtained.

$$ \boldsymbol{D}=\frac{\mathbf{NR}-\mathbf{NS}}{\mathbf{NS}} $$

D = difference between the redistributed deaths and those reported to SIM due to neoplasms, in relation to the number of deaths reported to SIM due to neoplasms; NR = number of deaths redistributed due to neoplasms; and NS = number of deaths reported to SIM due to neoplasms.

The difference obtained was added to the value 1 to calculate the correction factor, as the number 1 is a neutral factor in multiplications:

$$ F= 1+D $$

F = correction factor for chapter II (neoplasms); and D = difference between the redistributed deaths and those reported to SIM due to neoplasms, in relation to the number of deaths reported to SIM due to neoplasms.

This factor was multiplied by the number of cancer-related deaths. Therefore, it was considered that the correction factor for chapter II could be applicable to testicular cancer:

$$ DC=F\times NDS $$

DC = corrected number of deaths due to testicular malignant neoplasms), NDS = number of deaths reported to SIM due to testicular malignant neoplasms), and F.

With information on the readjusted number of deaths, standardized mortality rates were calculated, adjusted in accordance with the world population, per 100,000 men [11]. Population data by region and age group were obtained from demographic census information and inter-census predictions, from the website of the Brazilian Institute of Geography and Statistics [12].

Temporal mortality trends for testicular cancer were calculated for Brazil and its geographic regions, and mortality predictions were estimated until 2030, in five-year periods (2016–2020, 2021–2025 and 2026–2030).

Analysis of mortality trends employed Joinpoint regression, utilizing the software *Joinpoint Regression Program*, Version 4.4.0, 2017. The objective is to identify the occurrence of possible joinpoints, where significant changes in trends have occurred.

The method employed identified joinpoints based on the model with a maximum of three change points. The final selected model was the most adjusted model, with *Annual Percentage Change* (APC) based on the trend of each segment, estimating whether these values were statistically significant to a 0.05 level, to describe the terms “significant increase” or “significant decrease”. The significance levels utilized herein are based on the Monte Carlo permutation method and on the calculation of the annual percentage change of the ratio, utilizing the logarithm of the ratio [13].

Nordpred (Cancer Registry of Norway, Oslo, Norway), inscribed within statistical program R, was employed to calculate the predictions for each period, utilizing the age-period-cohort. Data were compiled in 5-year blocks and the limit age group considered for analysis was the first with more than 10 cases for the combined period.

Future rates were predicted, until 2030, using the average trend of all observed data (15-year cohorts). Observed and predicted rates for each five-year period were compiled for the population of men in the age groups of 0–14, 15–39 and over 40 years old. The projection of the recent linear trend for the last ten years was attenuated in the drift parameter of 25% in the second and 50% in third 5-year period prediction periods.

The results of the predictions are presented for the total of observed and expected deaths for each period, for Brazil and its five geographic regions. For each period, adjusted mortality rates based on the world standard population were calculated for global comparisons, expressed per 100,000 inhabitants/year (ASW∕100,000 inhab) [11].

Annual changes in the number of deaths in the last predicted period (2026–2030) were calculated in comparison with the last observed period (2011–2015), where the proportion of this change was verified in terms of changes in risks or demographics (size or structure of the population). These components can be different from zero and present a positive or negative direction. Calculation is expressed as [14].

$$ {\varDelta}_{tot}={\varDelta}_{risk}+{\varDelta}_{pop}=\left({N}_{fff}-{N}_{off}\right)+\left({N}_{off}-{N}_{ooo}\right) $$

*Δ*tot = total change, *Δ*risk = change in function of risk, *Δ*pop = change in function of the population, *Nooo* = number of observed cases, *Nfff* = number of predicted cases, Noff = number of expected cases when mortality cases increase during the observed period.

## Results

**Fig. 1:**
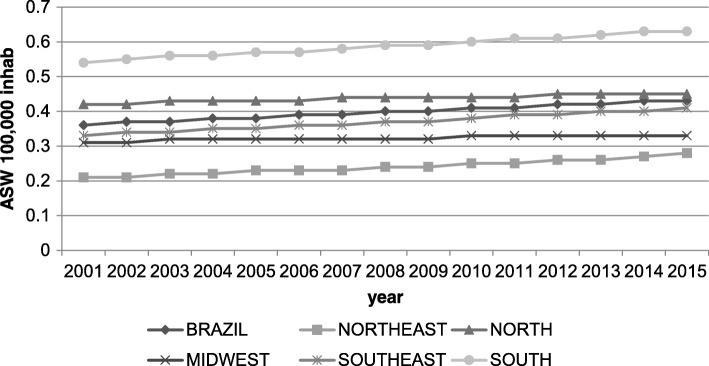
Standardized testicular mortality rates for Brazil and its geograpphic regions, for the period 2001–2015. Brazil, 2018

For the period 2001–2015, there were 4075 deaths due to malignant testicular neoplasm in Brazil. The mortality rate standardized to the world population varied between 0.36 deaths/100,000 men in 2001, to 0.41 deaths/100,000 men in 2015. Mortality rates higher than the overall Brazilian average were registered for the Southern and Northern regions of the country (Fig. 1). When analyzing the historical series of mortality rates, increasing trends were detected for Brazil (APC of 1.3%) and the Southeastern region (APC of 1.5%) (*p* < 0.05) with stability for the remaining geographic regions, with no joinpoints (Table 1).

**Table 1:** Temporal trends for testicular cancer mortality in Brazil and its geographic regions: number of deaths, *Annual Percentage Change* (APC), confidence interval and year of joinpoint occurrence, with the total number of deaths over the 15 years, for the period 2001–2015. Brazil, 2018

	Number of deaths	APC (CI 95%)	Joinpoint	Annual population size
Brazil	5996	1.3*(0.6; 2.0)	–	94.558.522
Northeast	957	2.0 (−0.1; 4.3)	–	
North	530	0.5 (−3.7; 4.8)	–	
Midwest	339	0.5 (−3.5; 4.6)	–	
Southeast	2441	1.5* (0.2; 2.7)	–	
South	1303	1.1 (−0.3; 2.4)	–	

*Statistical significance p < 0.05

*APC* Annual percentage change; 95% CI, 95% confidence interval

Table 2 presents the number of deaths and standardized mortality rates for the observed and predicted periods. Data analysis for the 5-year period 2026–2030 in Brazil predicted the occurrence of 2888 deaths due to testicular cancer. Predictions indicate that mortality is increasing in Brazil and the South, North and Northeast regions, while there is stability for the Southeast region and slight reduction in rates until 2030 for the Midwest.

**Table 2:** Testicular cancer mortality in Brazil: number of observed and projected deaths per age group and mortality rates adjusted to the world population (ASW/100,000 inhab)

	Observed			Projected		
	2001–2005	2006–2010	2011–2015	2016–2020	2021–2025	2026–2030
BRAZIL						
Age (years)						
0–14	22	18	22	18	17	16
15–39	1189	1520	1645	1817	1876	1896
≥ 40	472	494	614	697	831	976
ASW	0.36	0.39	0.41	0.44	0.45	0.47
Northeast						
Age (years)						
0–14	5	6	12	8	8	7
15–39	189	207	240	256	269	275
≥ 40	65	118	115	145	167	186
ASW	0.2	0.25	0.25	0.27	0.28	0.29
North						
Age (years)						
0–14	0	2	3	2	2	2
15–39	81	155	163	192	207	222
≥ 40	47	30	49	51	66	88
ASW	0.41	0.45	0.47	0.48	0.5	0.53
Midwest						
Age (anos)						
0–14	3	0	2	1	1	1
15–39	53	88	95	91	84	80
≥ 40	33	33	32	43	41	51
ASW	0.29	0.32	0.31	0.28	0.26	0.26
Southeast						
Age (years)						
0–14	9	6	3	4	4	4
15–39	478	649	668	706	699	671
≥ 40	190	196	242	257	297	350
ASW	0.33	0.38	0.38	0.39	0.39	0.39
South						
Age (years)						
0–14	3	3	1	2	2	2
15–39	249	279	349	429	472	496
≥ 40	129	120	170	231	313	394
ASW	0.55	0.53	0.63	0.77	0.87	0.96

**Fig. 2:**
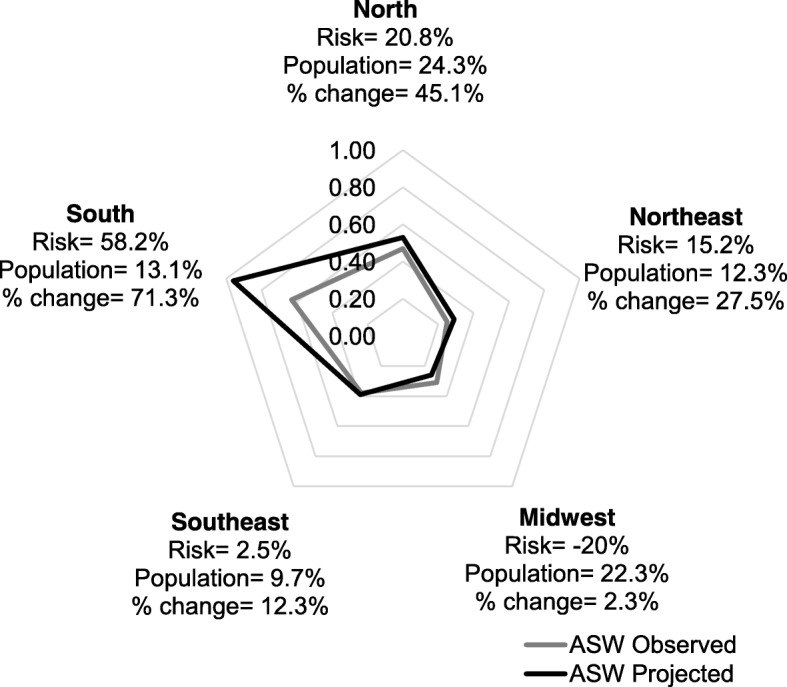
Testicular cancer mortality rates (ASW) (world standard) in Brazilian regions for 2001–2015 (observed period) and 2026–2030 (projected period), total change (change), change due to change in risk (risk), and change due to demographic alterations (population)

Figure 2 presents the mortality rates for testicular cancer, in the observed and estimated periods, according to the influence of risks and population structure of Brazil and its geographic regions. For Brazil, there will be a 26.6% increase in the number of deaths due to testicular cancer in the period 2026–2030 when compared with 2011–2015, and this change is explained mostly by an increase in the risk of death (14.2%), followed by modifications in the demographic structure of the country (12.4%). For the South region, predictions indicate an increase of 71.3% in the number of deaths, explained mostly by an increase in the risk of dying from the disease (58.2%). For the North region, the increase will be of 45.1%, explained by changes in demographic structure (24.3%) as well as by risk of death (20.8%).

## Discussion

This study revealed increasing trends for testicular cancer mortality in Brazil, and that until 2030, the mortality burden will continue to increase; for this period, a change of approximately 30% will be recorded in the number of deaths due to this disease. For this type of cancer, it is observed that in areas where high incidences were historically registered, there are actually decreasing trends. However, in most countries of Latin America and some parts of Asia, South Africa and southern Europe, testicular cancer starts to emerge as an important cause of morbidity and mortality in young men [15, 16].

In 2012, incidence rates for testicular cancer varied more than 44 times around the world. The highest rates were detected in Western Europe (8.7 cases/100,000 men), Northern Europe (7.2 cases/100,000 men) and Australia/New Zealand (6.8 cases/100,000 men), while the lowest rates were observed in Middle Africa (0.2 cases/100,000 men) [17]. The estimates for year 2018 show that the highest incidence rates remained in the same regions: Western Europe (9.7 cases/100,000 men), Northern Europe (7.2 cases/100,000 men) and Australia/New Zealand (7.4 cases/100,000 men), while the lowest rates were found in Middle Africa (7.2 cases/100,000 men) [18]. Incidence trends, within the last 10 years before 2012, showed persistent increases in the majority of countries of the world, except for China (APC = − 0.3%). The most pronounced increasing trends were registered in Southern Europe, with APC = 6.8% in Croatia and 6.1% in Spain [17]. This increasing trend for Southern Europe has already been present in the 10 years before 2008 [14].

In 2012, the highest mortality estimates of the world were found in Latin America/the Caribbean (0.55 deaths/ 100,000 men), followed by Europe (0.4 deaths/100,000 men) [17]. Estimated data for 2018 remained in the same perspective [1]. The highest value of the ASR-World mortality was reached in the estimates for 2018 in Chile and Georgia (1.1/100,000, both), followed by Niger (0.91/100,000), and Argentina (0.85/100,000). The lowest mortality rates were Dominican Republic and Singapore (0.02/100,000, both), and in many African and Asian countries [18]. The most recent trend, analyzing the 10 years before 2012, verified that most countries presented a reduction in mortality, especially China, which presented a significant decrease in mortality (APC = − 6.1%). Croatia, Brazil, USA and Colombia were exceptions, with increasing testicular cancer mortality (APC = 1.7, 1.8, 0.9 and 0.9%, respectively). Although Europe presents a secondary position regarding global mortality rates, these are still relatively low when compared with high incidence, differently from what is observed in regions of Africa and Asia, where mortality rates are close to the incidence rates [17].

As observed herein and when comparing global rates, Brazilian mortality rates that are lower than those of some developed countries and some Latin American countries, but with increasing predictions until 2030.

When evaluating the trends and predictions for testicular cancer mortality in Brazil, different patterns were identified across the geographic regions, demonstrating the epidemiological diversity of the country. The highest rates were registered in the South region, while the lowest rates were identified in the Northeast region. Trend analysis demonstrated that, in the last 15 years, there were significant increases in the overall Brazilian rates and for the Southeast region, with stable trends for the remaining regions. It must be highlighted that, although there were different patterns for mortality trends, the predictions indicated increases in the crude number of deaths for all regions. This fact is justified mainly by changes in the size and structure of the population. Predictions for 2030 indicate increasing mortality rates for Brazil and for the South, North and Northeast regions. Decreasing rates should occur for the Midwest region, with stability for the Southeast.

The worst social and economic conditions are concentrated in the North and Northeast regions of Brazil. The most developed regions of the country, South and Southeast, present better per capita income, lower illiteracy rate and overall better infrastructure of health services. The Midwest region presents an intermediate condition [19].

It is difficult to explain the geographic and temporal variations observed in population levels and translate these to the testicular cancer mortality rates in the absence of well-defined risk determinants. However, there is a direct relationship between mortality reduction and the organization of urological care services and the introduction and availability of cisplatin-based therapies in the 1970s. The reduction in mortality rates experienced by the majority of European countries in the most recent decades and in the United States was associated with improvements in diagnosis and efficacy of chemotherapy, with the inclusion of cisplatin in treatment regimes [20].

The epidemiological transition of Brazil has defined a picture of epidemiological polarization, where infecto-contagious diseases share space with an increase in non-communicable chronic diseases, such as cancer [21]. Besides, there is also significant inequity regarding access to cancer diagnosis and treatment services in large urban centers and in more interior locations, as occurs in other South American countries [22, 23]. In Brazil, there is a concentration of oncological services in large urban centers that present better socioeconomic levels (Southeast and South regions). This inequality in the distribution of services contributes to delays in diagnosis and treatment of patients in the least wealthy regions, which in the end present worse prognosis when compared with patients that live in urban centers. This also increases the pressure on medical reference centers due to the migration of patients to larger cities [21].

One of the causes related to cancer mortality in a defined population is the prevalence and distribution of related risk factors [24]. For testicular cancer, ethnicity appears as a risk factor for the increase of incidence and, consequently, affects mortality rates. In the USA, incidence is almost five times higher in Caucasians (9.1 cases per 100,000 men/year) than in Afrodescendants (2.0 cases per 100,000 men/year). In this aspect, the high mortality rates predicted for testicular cancer in the South region of Brazil could be partly explained by the phenotypic characteristics of that population. The South presents a predominance of European descendants, with a high proportion of Caucasians (more than 70% of the population), while the North and Northeast regions present Caucasian percentages under 40% [25, 26]. Another possible explanation for the mortality trends detected for the South region of Brazil could be the better structure of epidemiological cancer vigilance services, characteristic of well-developed regions, which results in better quality of death registries [23].

Nevertheless, the increasing rates observed for the poorest regions of Brazil could be a result of acquiescence and challenging access to the health service network in these areas [27], as prophylactic actions and availability and access to diagnosis and treatment methods affect cancer survival and mortality rates of a population [28].

Considering that 5-year localized testicular cancer survival is 99.2%, minimization of the impact that this type of cancer could cause in the future should count with special attention to the detection of early stage lesions [29]. The high level of survival of testicular cancer patients in the Slovak Republic in comparison with several other countries of eastern Europe with worse survival rates were probably influenced by the massive education of physicians of first examining the testis within the framework of preventive physical examinations [30]. Priority should be given to primary assistance within public health strategies through the implementation of primary prevention measures, and especially, of educational and active vigilance measures that favor self-examination. The advances and improvements in treatment techniques and protocols are very relevant, along with better access and availability to treatment in due time, and training of health professionals so that these can effectively promote health awareness and early detection of suspected cases [29].

A limitation of this study is the utilization of secondary data on mortality, which is subject to under-registry. However, considerable improvements have been attained recently regarding the quality of SIM in Brazil. This study applied data correction, which reduces this potential bias. Cancer predictions should be interpreted with caution, as diagnosis and therapeutic conditions can change in the future and, consequently, mortality trends could be slightly modified. However, the currently employed methods have certified validity, and some authors have pointed differences between 10 and 20% between estimates and registries of events [31].

## Conclusions

The findings of this study reveal increasing trends for testicular cancer mortality in Brazil for the 15 years analyzed, and these rates will continue to increase until 2030, also indicating that there are inequalities in the distribution of testicular cancer mortality across Brazilian regions. In this aspect, the phenotypic profile of the Brazilian population is considered along with inequalities in the offer and access to prevention, vigilance, diagnosis and treatment of cancer in Brazil. With the aim of reducing the possible disparities in testicular cancer mortality across Brazil, it is paramount to implement public health policies directed to the most affected populations, associated with a decrease in social inequalities and improvement of access to primary prevention, early diagnosis and effective treatments.

## Data Availability

Data were obtained from secondary data registered in the Mortality Information System (SIM) of the Informatics Department of the Unified Health System (Brazil’s publicly funded health system). Available at: (http://datasus.saude.gov.br/informacoes-de-saude/tabnet).

## Abbreviations

APC: Annual Percentage Change
BIGS: Brazilian Institute of Geography and Statistics
HDI: Human Development Indices
ICD-10: International Classification of Diseases, 10th Revision
SIM: Mortality Information System
